# Theoretical Assessment of Thermodynamic Stability in Nanocrystalline Metallic Alloys

**DOI:** 10.3390/ma12203408

**Published:** 2019-10-18

**Authors:** Antonio Mario Locci

**Affiliations:** Dipartimento di Ingegneria Meccanica, Chimica, e dei Materiali, Università degli Studi di Cagliari, via Marengo 2, 09123 Cagliari, Italy; antonio.locci@dimcm.unica.it

**Keywords:** modeling, thermodynamics, polycrystalline alloys, grain boundary segregation

## Abstract

Thermal stability in nanocrystalline alloys has been extensively explored while using both experimental and theoretical approaches. From the theoretical point of view, the vast majority of the models proposed in the literature have been implicitly limited to immiscible or dilute systems and thus lack the necessary generality to make predictions for different alloying interactions and in the case of intermetallic compounds formation. In this work, a general theoretical description for the case of binary W-based alloys is presented. It is shown that a critical value $\Omega^{*}$ of the interaction energy in the grain boundary $\Omega^{\left(gb\right)}$ exists, such that the condition $\Omega^{\left(gb\right)}<\Omega^{*}$ can be regarded as a criterion for thermodynamic stability assessment. A procedure for calculating the value of $\Omega^{*}$ for each specific alloy is illustrated. A preliminary qualitative comparison between the model predictions and properly selected experimental findings taken from the literature and related to the W-Cr system is also provided.

## 1. Introduction

It has long been a primary goal of materials scientists and engineers to design the structure of polycrystalline materials and to control the coarsening phenomena that can occur during their processing and applications, since a wide variety of materials properties is known to depend on the grain size and size distribution. Scientific and technological interest in this field has gained additional impulse from the development of nanostructured materials [1,2]. Unique features indeed appear as the structural characteristic length of polycrystalline materials is reduced from the micro-scale to the nanometer range. The increasing number of interfaces and their character play a central role in determining, and may eventually dominate, the overall properties of such fine grained polycrystals. Therefore, nanocrystalline materials have generated interest in terms of both the challenges that they pose to fundamental scientific understanding and the opportunity that they present for several conventional and emerging technological applications. This interest stems from the benefits that nanostructured materials may exhibit in terms of properties and potential applications that may not be achievable while using their coarse-grained counterparts [3,4,5].

Even though nanoscale grain sizes can be easily achieved in several polycrystalline materials, nanostructure stability is of primary concern during subsequent processing and service operation. Indeed, nanocrystalline materials tend to be unstable against grain coarsening due to their necessarily large volume fraction of grain boundaries (GBs), which have an associated energetic penalty with respect to the bulk (GB-free) reference state. The natural tendency of any system to seek a configuration that allows for the lowest possible energy introduces a large driving force for grain growth out of the desired nanocrystalline range. Materials may then evolve toward a lower-energy and coarser-grained state, such that nanostructure properties enhancement is lost. Therefore, the technological application of nanocrystalline materials crucially depends on the improvement of their stability through the development of effective methods to suppress or retard grain growth [2,5,6,7,8,9,10,11].

Investigators have considered various approaches to stabilize materials nanostructure due to the limitations described above. In particular, alloying has been the key of a significant enhancement of nanoscale materials stability by extending the temperature range where a nanostructure can be maintained. For instance, several nanocrystalline metallic alloys have demonstrated superior elevated temperature behavior as compared with their pure metal counterparts [5]. However, fundamental to the improvement of this successful stabilization strategy is an understanding of the factors governing the migration of the interfaces that define the polycrystalline material structure [2]. In this regard, it is well known that grain growth is expressed by the product of the grain-boundary mobility, M, and the driving force P [12]. Therefore, extended nanostructure stability that is induced by phenomena, such as segregation or secondary phase precipitation occurring at grain boundaries of multicomponent materials, is typically understood according to two different perspectives, i.e., kinetic or thermodynamic one. In the former case, GB segregated atoms or precipitated particles that are induced by alloying addition decreases grain boundary mobility by the solute-drag or Zener (particle) pinning effects, respectively [13,14]. On the other hand, the thermodynamic interpretation assumes that the improved stability might derive from a reduction of the driving force for grain growth [2,5].

It is worth mentioning that, where slowing grain boundary motion can be sufficient for some applications, the kinetic nature of grain boundary mobility often yields a merely transient stability of nanostructures. Indeed, GB mobility varies with temperature according to an Arrhenius law. Therefore, even a drastic reduction of M will be eventually overcome at high temperature and grain growth would restart accordingly [2,13]. In this sense, potentially more effective would be the thermodynamic approach, since the associated reduction of grain boundary energy through the segregation of solute atoms to the grain boundaries only exhibits weak temperature dependence [13]. Therefore, if alloying can eliminate the driving force for grain growth, nanocrystalline alloys can then be developed to retain their structure for a longer time at-temperature or at higher temperatures. This would provide a better control over nanostructure, and possibly a wider range of processing routes and applications. J. Weissmuller related the concept to nanocrystalline metallic alloys in a seminal paper describing thermodynamic nanostructure stabilization [3]. Conceptually, the basis for the decrease of GB energy in these systems relies on the inhomogeneous distribution of some of the alloys components, which tend to preferentially occupy grain boundary sites. The grain boundary energy is reduced in this segregated state, since some atoms can more effectively satisfy their bonding preference and/or reduce elastic mismatch strains in the partially disordered structure that typically characterize the intergranular region [5]. More recent studies have confirmed that nanocrystalline metallic alloys could, in fact, be thermodynamically stabilized by enrichment at the grain boundaries of some specific alloy component [15].

Although thermodynamic stability in nanocrystalline alloys has been extensively explored using both experimental and theoretical approaches, our basic understanding of this phenomenon remains incomplete and several issues are still required to be clarified. In particular, one outstanding question regarding nanostructure thermodynamic stability lies in the prediction of alloy systems that should display it. Analytical [3,7,13,16,17,18,19,20,21,22], atomistic [5,9,23,24,25,26,27], or statistical thermodynamics-based [6,28,29,30,31] models have been proposed to address this issue. However, regardless of the specific method adopted, the model prediction capability strongly relies on the availability of grain boundary parameters that are typically difficult to reliably measure or estimate. Indeed, the few successful attempts to relate modeling predictions to experiments have depended on the use of various fitting parameters. Therefore, theoretical predictions have achieved varying levels of success only on specific individual alloy systems, and they do not typically provide enough information to compare with and extrapolate to other alloying elements or to unexplored systems. Furthermore, the vast majority of models dealing with metallic alloys have been limited to immiscible or dilute systems, such that they cannot be used to make predictions for different alloying interactions (i.e., negative enthalpy of mixing) and in the case of intermetallic compounds formation.

In this work, a thermodynamic model is formulated without restricting ourselves to the dilute limit or to immiscible systems. The derivation is independent of structure geometries and it includes interactions among GB atoms. A novel methodology for the theoretical assessment of the thermodynamic stability in polycrystalline alloys according to the phase equilibria scheme and a general criterion for predicting stability against grain growth, as well as against phase separation and intermetallic compound formation, are also proposed. The resulting theoretical tool is applied to W-based systems and a comparative analysis of various binary alloys is provided.

## 2. Modeling of Thermodynamic Stability in Polycrystalline Substitutional Alloys

### 2.1. Model Equations

The thermodynamics of metallic alloys traditionally only focuses on material configurations that comprise “bulk” or crystalline regions, such as homogeneous solid solutions, phase-separated solid solutions, and intermetallic compounds. However, when the characteristic internal length scale of the system (such as the grain size) is reduced to the nanoscale, the interfaces become increasingly relevant and it can enable different configurations that are not explicitly accounted for in conventional bulk thermodynamics. Considering the presence or absence of such interfaces as an additional possible degree of freedom for the alloy configuration naturally connects grain boundary solute segregation to nano-system thermodynamics [9].

The mathematical description of the Gibbs free energy, *G* (J), is needed in order to predict thermodynamic stability in polycrystalline substitutional alloys as an effect of grain boundary segregation. The energetic contribution of grain boundaries is typically expressed by taking advantage of the surface energy concept. Indeed, according to Gibbs, J.W., the surface energy σ (J/m^2^) represents the difference between the Gibbs free energy of the actual system containing the surface *A* (m^2^) and the Gibbs free energy of a chosen reference system, which, of course has no surface [32]. Putting this sentence in formula:

(1) $$\sigma =\frac{G-G^{\left(*\right)}}{A}$$

where the superscript (*) has, hereafter, the meaning of reference quantity. As the reference system is homogeneous (i.e., it has no surface), its Gibbs free energy can be expressed, as follows:

(2) $$G^{\left(*\right)}=\overset{n}{\underset{i=1}{\sum }}\mu_{i}^{\left(*\right)}\left(x_{i}^{\left(*\right)}\right)N_{i}^{\left(*\right)}$$

where n is the number of chemical species composing the system, μ (J/mol) the chemical potential, x the molar fraction, and N (mol) the number of moles. The chemical potential dependence upon the composition is explicitly shown in Equation (2) for later considerations.

Substituting Equation (2) into Equation (1) and rearranging, the Gibbs free energy of the actual system can be expressed according to the following equation:

(3) $$G=\overset{n}{\underset{i=1}{\sum }}\mu_{i}^{\left(*\right)}\left(x_{i}^{\left(*\right)}\right)N_{i}^{\left(*\right)}+\sigma A$$

Equation (3) represents, in essence, the so-called “surface approach” to the thermodynamic description of heterogeneous systems. This equation can be regarded as general, since it does not pose any restriction regarding the choice of the reference system. On the other hand, the usefulness of Equation (3) strongly depends upon the availability of the surface energy assessment. Moreover, the choice of the σ expression is not unique or independent, because it must be in agreement with the chosen reference system (see Equation (1)).

The later issue does not represent a real problem in single-component systems, since the choice of the reference system is straightforward. On the other hand, in multi-component systems, the surface energy should also consider other contributions besides the simple surface formation. Indeed, chemical interaction between the components can be affected by the presence of surfaces. In addition, surface energy evaluation should also take into account the distinction between open and closed systems. For instance, surface adsorption can modify the surface composition as well as the total number of moles of the system, while keeping the grain interior composition constant. On the other hand, grain boundary segregation alters the composition of both the bulk (grain interior) and grain boundary, while keeping the total number of moles constant. In the case of the former phenomenon, the reference state might have the same number of moles of the original system (i.e., before adsorption) or, alternatively, the one resulting at end of this process. Similarly, in the case of grain boundary segregation, the reference system might have the global composition or the grain interior one resulting at the end of the atom redistribution phenomenon. It is then clear that the energetic contribution of the above-mentioned phenomena can be embedded in the surface energy term in different degrees, depending on the reference system choice (see Equations (1)–(3)).

It might be convenient to describe the Gibbs free energy of these systems by the so-called “surface phase approach” in order to shed some light on this issue for the case of polycrystalline materials. The starting point is the definition of phases as “different homogeneous bodies” that “differ in composition or state” [32]. More specifically, a phase can be regarded as a continuum of spatially homogeneous thermodynamic states that can be described while using a given fundamental equation. It follows that the above-mentioned generalized phase definition allows for describing grain boundary as a phase. It is worth also noting that a recent statistical thermodynamics-based model describes the GB as an intergranular region of finite volume with its own thermodynamic and structural properties, such as bond energies and composition [6]. It was therein assumed that composition, temperature, and pressure are homogeneous within both the grain interior and GB region. Such assumptions make the latter one fulfill the Gibbs meaning of phase, since, by definition, there exists a fundamental equation that describes GBs as a homogeneous body. Furthermore, considering GBs as a phase agrees with classical [33] and recent [34] literature.

**Figure 1:**
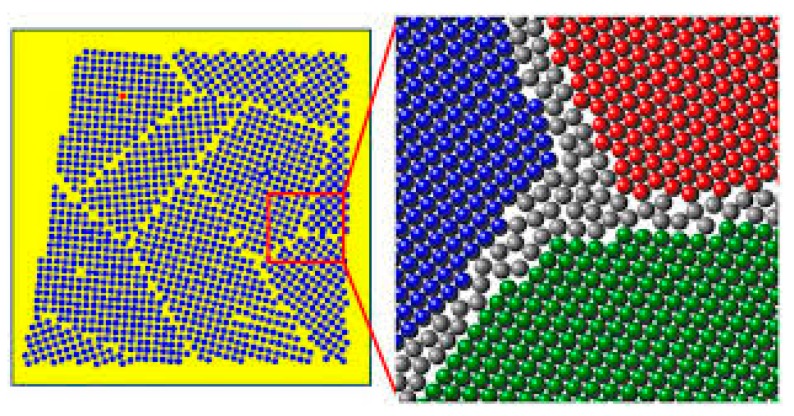
Representation of multi crystalline solutions (colored areas) system with intergranular regions of a finite volume (white area).

Therefore, in this work, it is assumed polycrystals as heterogeneous systems consisting of diverse grains (*g*) having equal size and composition. We also assume a common grain boundary region (*gb*) separates grains, as shown in Figure 1. According to the surface-phase approach, grain boundary is then mathematically treated as a volume (3D) phase (intergranular region), and is regarded as a homogeneous phase. This implies that its Gibbs free energy can be evaluated by using the same relations adopted for bulk systems. It is worth noting that, in this work, the term “bulk” and the symbol (*b*) will be used to identify the phases free from GBs, i.e., phases that are characterized by a grain size tending to infinite (d→∞). Additionally, no reference is made to geometric surfaces or interfaces separating the grain interior from the intergranular region. This allows for relating the entire energetic contribution of GBs to their volume, thus reducing the mathematical treatment complexity.

The Gibbs free energy of polycrystalline systems can be then expressed as

(4) $$G=G^{\left(g\right)}+G^{\left(gb\right)}$$

Equation (4) states that the Gibbs free energy of the system is the sum of the grains interior (*g*) and grain boundaries (*gb*) contributions. Expressing the Gibbs free energy as a function of the chemical potential, we have

(5) $$G=\overset{n}{\underset{i=1}{\sum }}\mu_{i}^{\left(g\right)}\left(x_{i}^{\left(g\right)}\right)N_{i}^{\left(g\right)}+\overset{n}{\underset{i=1}{\sum }}\mu_{i}^{\left(gb\right)}\left(x_{i}^{\left(gb\right)}\right)N_{i}^{\left(gb\right)}$$

or

(6) G=∑i=1n(μ0i(g)+RTlnai(g))Ni(g)+∑i=1n(μ0i(gb)+RTlnai(gb))Ni(gb)

where μ0i( ) and $a_{i}^{\left(\;\right)}$ are the chemical potential of the *i*-th component in its reference state and the activity of the same component, respectively, while R (J mol^−1^ K^−1^) and T (K) have the usual meaning of universal gas constant and temperature. It is worth noting that the activity of the same component in the grain interior and in the grain boundary are generally expected to be different due to the different coordination numbers and atomic distances in the respective cases [35]. This difference becomes of central importance in the case of nanostructured alloys due to their high density of interfaces. Indeed, the interaction between alloy atoms, also in GBs, strongly affect the properties of these solids [36].

While the chemical potential of the *i*-th component in its reference state for the grained phase (μ0i(g)) can be assumed to be equal to the bulk state one (μ0i(b)), the same quantity of the GB phase can be defined, as follows:

(7) μ0i(gb)=μ0i(b)+a^iσi(gb)

where ${\hat{a}}_{i}$ is the molar surface of the *i*-th component belonging to the grain boundary, while $\sigma_{i}^{\left(gb\right)}$ is the surface energy of the pure component in the same GB phase. It will be clarified in the next that these quantities do not necessarily equal the corresponding ones in the reference (pure component) state, i.e., ${\hat{a}}_{i}^{0}$ and $\sigma_{i}^{0}$.

Substituting Equation (7) into Equation (6) and while using the assumption of closed systems (i.e., $N_{i}=N_{i}^{\left(b\right)}+N_{i}^{\left(gb\right)}$), the following expression can be obtained

(8) $$G=G^{0}+\overset{n}{\underset{i=1}{\sum }}\left(RTlna_{i}^{\left(g\right)}N_{i}^{\left(g\right)}\right)+\overset{n}{\underset{i=1}{\sum }}\left(RTlna_{i}^{\left(gb\right)}N_{i}^{\left(gb\right)}\right)+\overset{n}{\underset{i=1}{\sum }}\left({\hat{a}}_{i}\sigma_{i}^{\left(gb\right)}N_{i}^{\left(gb\right)}\right)$$

where the first term on the right-hand-side of Equation (8) is the Gibbs free energy of the material in its reference (GB-free and unmixed) state. Obviously, the sum of last three terms on the right-hand-side of Equation (8) represents the system Gibbs free energy of mixing:

(9) $$\Delta G_{mix}=\overset{n}{\underset{i=1}{\sum }}\left(RTlna_{i}^{\left(g\right)}N_{i}^{\left(g\right)}\right)+\overset{n}{\underset{i=1}{\sum }}\left(RTlna_{i}^{\left(gb\right)}N_{i}^{\left(gb\right)}\right)+\overset{n}{\underset{i=1}{\sum }}\left({\hat{a}}_{i}\sigma_{i}^{\left(gb\right)}N_{i}^{\left(gb\right)}\right)$$

To deal with the molar Gibbs free energy of mixing, let us rearrange Equation (9) by also adopting the usual expression of activity:

(10) $$\Delta G_{mix}=RT\overset{n}{\underset{i=1}{\sum }}\left[ln\left(\gamma_{i}^{\left(g\right)}x_{i}^{\left(g\right)}\right)x_{i}^{\left(g\right)}\right]N^{\left(g\right)}+RT\overset{n}{\underset{i=1}{\sum }}\left[ln\left(\gamma_{i}^{\left(gb\right)}x_{i}^{\left(gb\right)}\right)x_{i}^{\left(gb\right)}\right]N^{\left(gb\right)}+\overset{n}{\underset{i=1}{\sum }}\left({\hat{a}}_{i}\sigma_{i}^{\left(gb\right)}x_{i}^{\left(gb\right)}\right)N^{\left(g\right)}$$

where $\gamma_{i}^{\left(\;\right)}$ is the activity coefficient of the *i*-th component. Dividing by the total number of moles, N, of the system, the molar Gibbs free energy of mixing can be obtained:

(11) $$\Delta {\hat{g}}_{mix}=RT\overset{n}{\underset{i=1}{\sum }}\left[ln\left(\gamma_{i}^{\left(g\right)}x_{i}^{\left(g\right)}\right)x_{i}^{\left(g\right)}\right]\theta^{\left(g\right)}+RT\overset{n}{\underset{i=1}{\sum }}\left[ln\left(\gamma_{i}^{\left(gb\right)}x_{i}^{\left(gb\right)}\right)x_{i}^{\left(gb\right)}\right]\theta^{\left(gb\right)}+\overset{n}{\underset{i=1}{\sum }}\left({\hat{a}}_{i}\sigma_{i}^{\left(gb\right)}x_{i}^{\left(gb\right)}\right)\theta^{\left(gb\right)}$$

with $\theta^{\left(g\right)}$ and $\theta^{\left(gb\right)}$ the molar fraction of the grained and grain boundary phases, respectively.

In the following, the analysis will be limited to binary substitutional alloys that are described by the regular solution model, where *A* and *B* hereafter indicate the solvent and the solute, respectively. Accordingly,

(12) $$RTln\left(\gamma_{i}^{\left(j\right)}\right)=\Omega^{\left(j\right)}{\left(x_{k}^{\left(j\right)}\right)}^{2}\;\;\;\;j=g,\;gb;i=A,B;k\neq i=A,B$$

where $\Omega^{\left(j\right)}$ represents the interaction energy between A and B components in the *j*-th phase of the system.

Substituting Equations (12) into Equation (11) and rearranging the molar Gibbs free energy of mixing can be expressed as

(13) $$\Delta {\hat{g}}_{mix}=\Delta {\hat{g}}_{mix}^{\left(b\right)}\theta^{\left(g\right)}+\Delta {\hat{g}}_{mix}^{\left(gb\right)}\theta^{\left(gb\right)}$$

where

(14) $$\Delta {\hat{g}}_{mix}^{\left(b\right)}=RT\left(x_{A}^{\left(g\right)}lnx_{A}^{\left(g\right)}+x_{B}^{\left(g\right)}lnx_{B}^{\left(g\right)}\right)+\Omega^{\left(g\right)}x_{A}^{\left(g\right)}x_{B}^{\left(g\right)}$$

and

(15) $$\Delta {\hat{g}}_{mix}^{\left(gb\right)}=RT\left(x_{A}^{\left(gb\right)}lnx_{A}^{\left(gb\right)}+x_{B}^{\left(gb\right)}lnx_{B}^{\left(gb\right)}\right)+\Omega^{\left(gb\right)}x_{A}^{\left(gb\right)}x_{B}^{\left(gb\right)}+\left({\hat{a}}_{A}\sigma_{A}^{\left(gb\right)}x_{A}^{\left(gb\right)}+{\hat{a}}_{B}\sigma_{B}^{\left(gb\right)}x_{B}^{\left(gb\right)}\right)$$

represent the molar Gibbs free energy of mixing in the grained and grain boundary phases, respectively.

The following material balance relates the molar fractions of components in grained and grain boundary phases:

(16) $$x_{i}^{\left(g\right)}\theta^{\left(g\right)}+x_{i}^{\left(gb\right)}\theta^{\left(gb\right)}=x_{i}^{0}\;\;\;i=A,B;$$

where $x_{i}^{0}$ is the global molar fraction of the *i*-th component.

It might be clearly observed that grain size does not explicitly appear in the Gibbs free energy of mixing described by Equations (13)–(15). Indeed, the grain sizes could be evaluated through the relationship between them and the volumetric fractions, $f^{\left(\;\right)}$, of the phases composing the system. Latter ones are in turn related to the phase molar fraction, $\theta^{\left(\;\right)}$, as follows:

(17) $$f^{\left(l\right)}=\frac{\theta^{\left(l\right)}\sum_{i=1}^{n}{\bar{V}}_{i}^{\left(l\right)}x_{i}^{\left(l\right)}}{\theta^{\left(g\right)}\sum_{i=1}^{n}{\bar{V}}_{i}^{\left(g\right)}x_{i}^{\left(g\right)}+\theta^{\left(gb\right)}\sum_{i=1}^{n}{\bar{V}}_{i}^{\left(gb\right)}x_{i}^{\left(gb\right)}};\;l=g,gb$$

It turns out that phases volumetric and molar fractions are only equal when the partial molar volume ${\bar{V}}_{i}^{\left(\;\right)}$ of components are all equal in both phases. On the other hand, the relationship between volumetric fraction and grain size is strongly dependent upon the adopted geometric description of the system structure. For instance, Trelewicz and Schuh introduced the following relation between diameter and volumetric fraction of the grain boundary [6]:

(18) $$f^{\left(gb\right)}=1-{\left(\frac{d^{\left(g\right)}-t}{d^{\left(g\right)}}\right)}^{3}$$

where t represents the GB thickness according to the selected structure geometry and $d^{\left(g\right)}$ is the grain size. However, while Equation (18) properly described the case of single-particle systems, its extension to the case of multi-grained materials structure is not straightforward. In addition, Figure 1 shows that the GB thickness cannot be easily defined for the structure that is modelled in this work. It should be also considered that, according to the proposed model, phase equilibria and thermodynamic stability do not depend upon the specific $f^{\left(gb\right)}-d^{\left(g\right)}$ relation. Indeed, all of the thermodynamic quantities are defined as homogeneous first-degree functions of extensive (additive) variables (e.g., volume or number of moles). For these reasons, phase molar fractions are the only structural variables that are considered in this work, while their relation with the grain size will be addressed in a future work. It is worth mentioning that Equations (13)–(15) are basically identical to the model equations that are derived by Trelewicz and Schuh in a different way [6]. For the sake of completeness, it should be noted that the model aforementioned also postulates the existence of a transition surface between each grain interior and the intergranular region. However, their equations reduce to Equations (13)–(15) when neglecting the energetic contribution of this transition surface.

### 2.2. Model Parameters

Databases with model parameters are needed to investigate the thermodynamic stability of polycrystalline W alloys according to the model equations that are presented in the previous paragraph. Specifically, the grain boundary energy, $\sigma_{i}^{\left(gb\right)}$, and the grain boundary molar surface, ${\hat{a}}_{i}$, of components, as well as the interaction energy in the grained phase, $\Omega^{\left(g\right)}$, and the interaction energy in the grain boundary, $\Omega^{\left(gb\right)}$ should be provided or estimated. The GB energy of pure component is a complex function of temperature, pressure, and degrees of freedom of the given GB [37]. However, the following relation gives a reasonable estimation:

(19) $$\sigma_{i}^{\left(gb\right)}=\frac{1}{3}\sigma_{i}^{0}$$

where $\sigma_{i}^{0}$ is the surface energy of pure component in contact with vacuum [38].

Regarding the molar grain boundary surface associated to each component, ${\hat{a}}_{i}$, it should be noted that a GB atom only has a fraction of its surface in contact or belonging to the grain boundary. According to some authors, this fraction can be one-third of the molar surface of that component in its reference (pure) state, i.e., ${\hat{a}}_{i}=\frac{1}{3}{\hat{a}}_{i}^{0}$ [39]. Latter quantity can be related to the molar volume of pure component, according to the following relation [34]:

(20) $${\hat{a}}_{i}^{0}=f{\left({\hat{v}}_{i}^{0}\right)}^{\left(2/3\right)}N_{Av}^{\left(1/3\right)}$$

where $N_{Av}$ is the Avogadro number and f a coefficient typically ranging in the interval 1–1.25 [34,37]. Combining the information reported above, it can be obtained that

(21) $${\hat{a}}_{i}=\frac{{\hat{v}}_{i}^{0}}{\zeta^{*}}$$

where $\zeta^{*}$ may be regarded as the pure component GB characteristic thickness, given by

(22) $$\zeta^{*}=\frac{3}{f}{\left(\frac{{\hat{v}}_{i}^{0}}{N_{Av}}\right)}^{1/3}$$

Some authors assume a constant value of this parameter, i.e., $\zeta^{*}$ equal to 0.5 nm [6], while in this work it ranges from 0.60 nm for Be to 1.46 nm in the case of Cs. The molar surface energy of pure components at the grain boundary $\left(={\hat{a}}_{i}\sigma_{i}^{\left(gb\right)}\right)$ can be then estimated in the range 1.5–15.5 kJ/mol (see Table 1). Values of $\sigma_{i}^{0}$ and ${\hat{v}}_{i}^{0}$ provided by Bakker were used for these calculations [40]. The magnitudes of these values are in agreement with the ones reported by other authors, i.e., 8.25 kJ/mol [41]. However, it should be mentioned that the same authors assume that this value does not depend upon the specific alloys to be investigated. In this work, the molar surface energy of pure components is also multiplied by a factor equal to 0.71 to take the surface relaxation effect into account [39].

Parameter $\Omega^{\left(g\right)}$ can be reasonably assumed to be equal to the interaction energy in the bulk, $\Omega^{\left(b\right)}$, whose estimation is a relatively affordable task. Among several others, the method that was developed by Miedema and coworkers was adopted in this work [40,42,43]. According to this approach, the excess molar enthalpy of mixing can be expressed, as follows

(23) $$\Delta {\hat{h}}_{mix}^{\left(EX\right)}=x_{A}x_{B}\left(x_{A}^{s}\Delta {\hat{h}}_{B\to A}^{\left(chem\right)}+x_{B}^{s}\Delta {\hat{h}}_{A\to B}^{\left(chem\right)}+x_{A}\Delta {\hat{h}}_{B\to A}^{\left(elast\right)}+x_{B}\Delta {\hat{h}}_{A\to B}^{\left(elast\right)}+x_{A}\Delta {\hat{h}}_{B\to A}^{\left(struct\right)}+x_{B}\Delta {\hat{h}}_{A\to B}^{\left(struct\right)}\right)$$

where $x_{i}^{s}$ is the so-called surface concentration of the *i*-th component. $\Delta {\hat{h}}_{j\to i}^{\left(chem\right)}$, $\Delta {\hat{h}}_{j\to i}^{\left(elast\right)}$, and $\Delta {\hat{h}}_{j\to i}^{\left(struct\right)}$ are the chemical, elastic, and structural contributions to the molar enthalpy of solution of the solute *j* in the solvent *i* [40]. It can be clearly seen that Equation (23) does not fit the regular solution approximation Equations (13)–(15) are based on. On the other hand, since this work is limited to W-based (rich) alloys, it might be reasonable to assume that $\Delta {\hat{h}}_{B\to W}^{\left(\;\right)}\approx \Delta {\hat{h}}_{W\to B}^{\left(\;\right)}$ for the three contributions, being in this case the solute indicated by B. According to this assumption, Equation (23) can rewritten as:

(24) $$\Delta {\hat{h}}_{mix}^{\left(EX\right)}=x_{A}x_{B}\Omega^{\left(b\right)}$$

being the interaction energy now expressed as

(25) $$\Omega^{\left(b\right)}=\Delta {\hat{h}}_{B\to A}^{\left(chem\right)}+\Delta {\hat{h}}_{B\to A}^{\left(elast\right)}+\Delta {\hat{h}}_{B\to A}^{\left(struct\right)}$$

As far as the evaluation of interaction energy in bulk systems, $\Omega^{\left(b\right)}$, is concerned, the calculation of the first two terms appearing in the right-hand-side of Equation (25) is a well-established procedure and its details can be found elsewhere [40,42,43]. Table 1 reports the values of $\Delta {\hat{h}}_{B\to A}^{\left(chem\right)}$ and $\Delta {\hat{h}}_{B\to A}^{\left(elast\right)}$ for all the W-based binary alloys investigated in this work. On the other hand, the structural contribution $\Delta {\hat{h}}_{B\to A}^{\left(struct\right)}$ deserves additional comments. It can be written as [40,44]

(26) $$\Delta {\hat{h}}_{B\to A}^{\left(struct\right)}=\Delta E^{REF}+\left(Z_{A}-Z_{B}\right){\frac{dE_{A}^{REF}}{dZ}|}_{Z=Z_{B}}$$

where $\Delta E^{REF}=E_{A}^{REF}-E_{B}^{REF}$ and $E_{i}^{REF}$ is the lattice energy of the *i*-th element in its reference state. $\Delta E^{REF}$ can be easily evaluated according to the data for pure elements [45], and the corresponding values for all of the investigated W binary alloys are reported in Table 1. Regardless of the second contribution on the right-hand-side of Equation (26), it was originally evaluated only for 4d and 5d transition metals [46]. This because, functionality, or at least a trend, of the curve $E_{A}^{REF}$ versus Z is needed to evaluate the slope $\frac{dE_{A}^{REF}}{dZ}$. Even if this curve is known, Equation (26) could only be strictly applied to alloys of elements that are adjacent in the periodic table and belonging to the same period. Although some effort to increase the applicability of this method by overcoming the above-mentioned limitation has been reported [47], a thorough and reliable evaluation of $\Delta {\hat{h}}_{B\to A}^{\left(struct\right)}$ in the framework of the Miedema’s model is still an open issue. Therefore, it is assumed in this work that the contribution of the second term on the right-hand-side of Equation (26) can be neglected.

As far as the interaction energy in the grain boundary is concerned, widely accepted and reliable methods to estimate $\Omega^{\left(gb\right)}$ nowadays are not yet available in the literature. This is not surprising, because it is strongly related to the grain boundary structure, whose knowledge and comprehension is far to be exhaustive and still represent an open and challenging issue [48]. Therefore, in this work $\Omega^{\left(gb\right)}$ is related to $\Omega^{\left(b\right)}$, as follows:

(27) $$\Omega^{\left(gb\right)}=\beta \Omega^{\left(b\right)}$$

where the coefficient β is taken as a free parameter. This way, the fundamental role that is played by $\Omega^{\left(gb\right)}$ in determining the thermodynamic stability of polycrystalline materials can be highlighted.

## 3. Results and Discussion

Possible stable (equilibrium) states need to be identified to analyze the thermodynamic stability of polycrystalline solid solutions. According to expression of the molar Gibbs free energy of mixing given by Equations (13)–(15), there are three possible states, two mono-phasic, and one bi-phasic. Mono-phasic states are represented by systems where the bulk phase (*b*) or the intergranular regions (*gb*) only is present. States where the grained (*g*) and the intergranular regions coexist correspond to the latter one. The bulk phase ($\theta^{\left(g\right)}=1$) corresponds to the single crystal condition and does not need any additional comment. Instead, the other two possible states deserve further discussion.

Schuh and co-workers already hypothesized system states where only the grain boundary region is present ($\theta^{\left(gb\right)}=1$) [6,41]. Although the physical counterpart of this theoretical outcome still need to be better identified, it should be pointed out that it logically derives from the mathematical formalism adopted. Most interesting, the existence of the intergranular region is a necessary condition for defining the third possible state of the system, being defined by the coexistence of the grained and GB phases. This state can be identified by the condition $0<\theta^{\left(gb\right)}<1$, which, regardless of the specific relationship between molar and volumetric fractions and grain size, entails that the GB phase is present and the grain size has finite value.

The last thermodynamic state described above clearly identifies the polycrystalline system. It is then clear that, according to the formalism that was adopted in this work, polycrystalline structures are not single-phase systems, but rather the result of the co-existence of grained phase(s) and the intergranular (GB) region. It should be pointed out that Trelewicz and Schuh used the same conditions in their seminal paper to identify the nanostructured state [6]. However, as previously mentioned, a relationship between volumetric fraction and grain size should be provided to make a discrimination between coarse polycrystalline and nanocrystalline structures based on grain size. For instance, a nanocrystalline system is typically defined as a structure having grain size in the range between 0.5 and 100 nm. While using Equation (19) with t=0.5 nm, the $f^{\left(gb\right)}$ domain can be subdivided into the two intervals $0<f^{\left(gb\right)}\leq 0.015$ and $0.015<f^{\left(gb\right)}<1$. Whereas, the former interval represents the stability region of coarser structures, the second one identifies the stability range of nanostructures. However, in this work, this distinction will not be considered and the thermodynamic stability of polycrystalline systems will be investigated, regardless of the actual grain size. In this sense, the appearance of polycrystalline structure as the stable state could be considered a *conditio sine qua non* for the thermodynamic stability of nanostructures.

**Figure 2:**
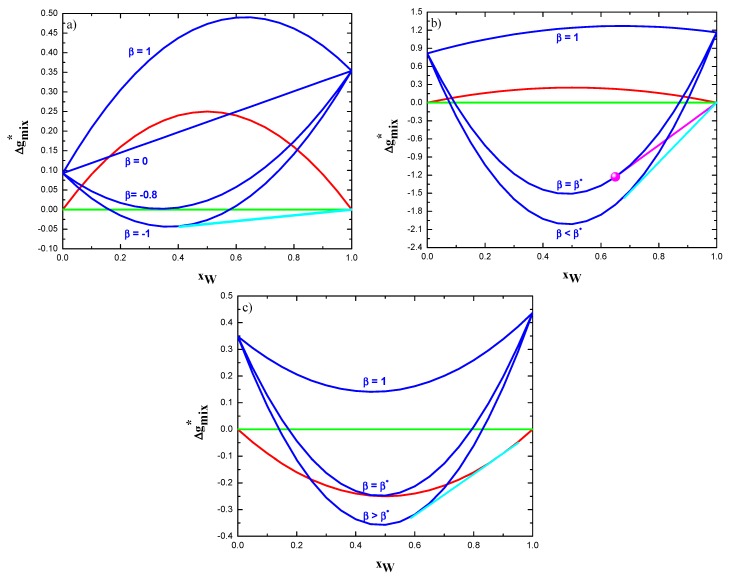
Phase equilibria of polycrystalline alloys: (**a**) W-Zn; (**b**) W-Zr; and, (**c**) W-Nb. Dimensionless Gibbs free energy as function of composition are reported for bulk solid solution (red), phase separated state (green), as well as grain boundary phase (blue). Latter one is depicted for different values of the coefficient β. Polycrystalline stable states are given by the common tangent (cyan) between grain boundary phase and bulk solid solution. Purple common tangent describes instead the existence of intermetallic-containing states. The point (magenta) characterizing the stoichiometric intermetallic compound W_2_Zr is also shown in figure (b).

Once temperature and global composition are given, calculating and comparing the Gibbs free energy of mixing of all accessible states identifies the equilibrium state of the system. The methodology that was adopted in this work is illustrated in Figure 2, where the dimensionless molar Gibbs free energy of mixing, $\Delta {\hat{g}}_{mix}^{*}$ ($=\Delta {\hat{g}}_{mix}/\Omega^{\left(b\right)})$ at 0 *K* of both bulk (see Equation (14)) and intergranular phases (see Equation (15) are plotted as a function of the global composition. For the sake of exemplification, data related to the W-Zn are used in Figure 2a. Therein, it can be seen that the molar Gibbs free energy of mixing of the grain boundary phase, $\Delta {\hat{g}}_{mix}^{\left(gb\right)}$, (blue curve) is higher that the bulk phase one, $\Delta {\hat{g}}_{mix}^{\left(b\right)}$, (red curve) whatever the alloy composition when β=1. Under these conditions, the polycrystalline structure is thermodynamically unstable when compared to the bulk solid solution. Decreasing the value of β (down to 0 in Figure 2a) a compositional range where the $\Delta {\hat{g}}_{mix}^{\left(gb\right)}<\Delta {\hat{g}}_{mix}^{\left(b\right)}$ appears. However, this is clearly a condition of metastability. Indeed, as $\Omega^{\left(b\right)}>0$ for the W-Zn alloy (see Table 1), the phase separated system (green line) represents the thermodynamically most stable state, whose molar Gibbs free energy of mixing, $\Delta {\hat{g}}_{mix}^{\left(\alpha \beta \right)}$, is zero at 0 *K*. It is worth specifying that this latter state is given by the co-existence of two bulk (GB-free) solid solutions.

By further decreasing the coefficient β, a value (β=−0.8 in Figure 2a) will be reached, such that there is at least one composition whose corresponding molar Gibbs free energy of mixing of the grain boundary phase is zero, i.e., $\Delta {\hat{g}}_{mix}^{\left(gb\right)}=\Delta {\hat{g}}_{mix}^{\left(\alpha \beta \right)}$. Let us identify this value as $\beta^{*}$ and name it as the critical β coefficient. It can be then seen that a common tangent (cyan line) between the pure tungsten phase and the grain boundary phase can be drawn when the condition $\beta <\beta^{*}$ is satisfied. Of course, the common tangent between the pure zinc phase and the grain boundary phase can be also drawn. However, it will be not here considered, since this work focuses on W-rich alloys. According to classical phase equilibria thermodynamics, such a tangent represents bi-phasic stable states that are characterized by the coexistence of pure W grained (*g*) phase and intergranular (*gb*) one. It is then apparent that $\beta <\beta^{*}$ identifies the necessary condition for polycrystalline structures to have thermodynamic stability.

The same analysis illustrated in Figure 2a can be extended to binary systems that are characterized by a positive value of $\Omega^{\left(b\right)}$ and forming intermetallic compounds. Figure 2b shows the case of W-Zr alloys at 0 K. Molar Gibbs free energy of bulk (red curve) and grain boundary (blue curves) phase, as well as of the separated phases state (green line), are reported according to the procedure already illustrated in Figure 2a. In addition, the point (magenta) characterizing the stoichiometric intermetallic compound W_2_Zr is also shown. The molar Gibbs free energy of this compound, $\Delta {\hat{g}}_{form}^{\left(\gamma \right)}$, was taken to be equal to −11.63 kJ/mol, as calculated by the Miedema’s method [40]. The purple line represents the common tangent between the intermetallic phase and the pure tungsten one. Therefore, it can be seen that, for the case of β=1, the stable states in the W-rich side are all a mixture of the just mentioned phases. This situation remains unaltered until the value of the coefficient β is decreased down to the critical value ($\beta =\beta^{*})$, which is equal to −9.99 per the case that is represented in Figure 2b. Indeed, in such a situation, the molar Gibbs free energy of mixing of the grain boundary phase with the same composition of W_2_Zr equals the molar Gibbs free energy of formation of this compound. Consequently, the critical parameter $\beta^{*}$ can be defined as the value of the coefficient β, such that $\Delta {\hat{g}}_{mix}^{\left(gb\right)}\left(x^{\gamma }\right)=\Delta {\hat{g}}_{form}^{\left(\gamma \right)}$, where $x^{\gamma }$ represents the stoichiometric composition of the intermetallic phase γ.

A parallel investigation can be performed for those systems showing negative interaction energy in the bulk, i.e., $\Omega^{\left(b\right)}<0$. As an example of these alloys, Figure 2c illustrates the thermodynamic phase equilibria taking place in the W-Nb system. A critical value of the coefficient β can also be identified for this category of alloys. However, in this case, $\beta^{*}$ ascertains the condition $\Delta {\hat{g}}_{mix}^{\left(gb\right)}=\Delta {\hat{g}}_{mix}^{\left(b\right)}$ for at least one composition of this alloy. It can also be observed that the polycrystalline structure is thermodynamically stable when the criteria ($\beta >\beta^{*})$ are fulfilled. The compositional range of these stable alloys is given in Figure 2c by the common tangent (cyan line), which represents the coexistence of the grain boundary phase (blue curve) and the grained solid solution (red curve).

**Figure 3:**
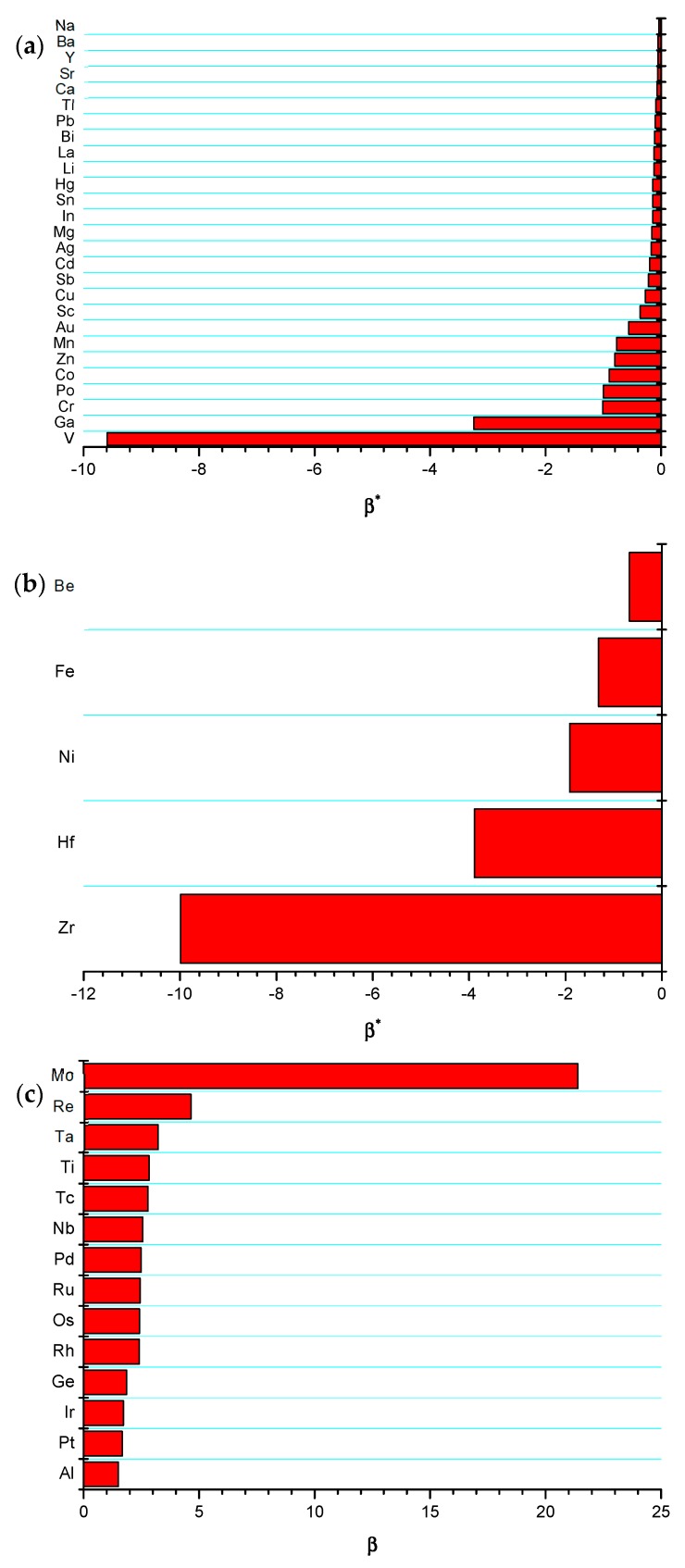
Value of the critical coefficient $\beta^{*}$ for W-alloys of group: (**a**) I; (**b**) II; and, (**c**) III.

For the sake of general comparison, the critical coefficient $\beta^{*}$ has been calculated following the procedure that is illustrated in Figure 2 for all possible W-based metallic alloys. The model results are grouped, as follows: (I) systems with $\Omega^{\left(b\right)}>0$ (Figure 3a); (II) systems with $\Omega^{\left(b\right)}>0$ forming intermetallic compounds (Figure 3b); and, (III) systems with $\Omega^{\left(b\right)}<0$ (Figure 3c). It is worth mentioning that a fourth group should be added, i.e., systems with $\Omega^{\left(b\right)}<0$ forming intermetallic compounds. However, among W alloys, only W-Al undoubtedly belongs to this group. Indeed, the existence of intermetallics at 0 K in system W-X (X = Ge, Ir, Os, Re, Rh, Ru) is largely uncertain. Latter ones have been then analyzed according to group III rules, i.e., neglecting the formation of intermetallic phases. Regarding the system W-Al, it will be investigated in detail in a forthcoming publication, while, in this work, a preliminary analysis of this alloy is given by neglecting the formation of WAl_4_, i.e., group III. It should be mentioned that this classification is based on data that are reported in Table 1.

A general overview of Figure 3a shows that the critical coefficients $\beta^{*}$ are all negative, with no exceptions. The same finding can be observed in Figure 3b, thus indicating that the effect of β in these alloys is dictated by the sign of $\Omega^{\left(b\right)}$. The intermetallic formation certainly affects the magnitude of $\beta^{*}$, but not its sign. Vice versa, a positive and greater that one value of $\beta^{*}$ (again with no exceptions) is revealed for all systems belonging to group III (see Figure 3c). It can easily demonstrated (see Equation (27)) that the two stability criteria ($\beta <\beta^{*}$ for groups I and II, and $\beta >\beta^{*}$ for group III), along the results that are reported in Figure 2 can also be translated into $\Omega^{\left(gb\right)}<\Omega^{*},$ where $\Omega^{*}$ can be regarded as a critical value for thermodynamic stability occurrence. The latter parameter should satisfy the following conditions: $\Omega^{*}<\Omega^{\left(b\right)}$ and $\Omega^{*}<0$. It is important to highlight that both conditions do not depend on the sign of $\Omega^{\left(b\right)}$ and that they should be simultaneously fulfilled.

This general conclusion can be understood, as follows. It is well known that GB formation always entails an energy penalty. In our model, such a penalty is represented by the term ${\hat{a}}_{i}\sigma_{i}^{\left(gb\right)}$ (see Equation (15)), which always shows positive values. Segregation occurs in the attempt of reducing the energy penalty in GBs by replacing the atoms characterized by higher ${\hat{a}}_{i}\sigma_{i}^{\left(gb\right)}$ value with those with a lower ${\hat{a}}_{i}\sigma_{i}^{\left(gb\right)}$ value. When these latter atoms saturate GBs, the polycrystalline structure still has Gibbs free energy that is higher than the bulk configuration. Therefore, latter state will be still preferred due to the natural tendency of the system to the lowest possible energy. This means that a polycrystalline system containing GBs could only exhibit thermodynamic stability if the energy penalty that is associated with GBs formation is balanced by something else.

Within the model proposed in this work, chemical mixing in the intergranular region (GB) can assure the requested balancing. Indeed, it is assumed that mixing energy in GB region, $\Omega^{\left(gb\right)}$, can be different from the bulk one, $\Omega^{\left(b\right)}$. Thus, when $\Omega^{\left(gb\right)}$ is smaller than $\Omega^{\left(b\right)}$, GB segregation is accompanied by a reduction of the mixing energy, which could be potentially able to compensate the energy penalty associated with GBs formation. However, thermodynamic stability depends on the relative magnitude of the two contributions. Indeed, the interaction energy $\Omega^{\left(gb\right)}$ should be “sufficiently smaller” than $\Omega^{\left(b\right)}$, such that the reduction of mixing enthalpy in the grain boundary overcomes the energy penalty due to GBs formation. Moreover, it has also been shown that the condition $\Omega^{\left(gb\right)}<\Omega^{\left(b\right)}$ is not enough to guarantee the thermodynamic stability of the alloys, also $\Omega^{\left(gb\right)}<0$ being a necessary condition. To explain this criterion, let us assume that grain boundary segregation takes place and that, consequently, the Gibbs free energy of the system is lowered with respect to the bulk state. However, this cannot be considered, yet the system preferred state if $\Omega^{\left(gb\right)}$ is positive. Indeed, $\Omega^{\left(gb\right)}>0$ means that components still do not “like” to stay together. Therefore, secondary phase precipitation is likely to occur at the grain boundary, such that components can complete their separation and further lower the Gibbs free energy of the system. On the other hand, $\Omega^{\left(gb\right)}<0$ means that the elements “like” to stay mixed in the grain boundary, thus making GB the preferred condition once the surface energy increase is compensated by the reduction of the mixing energy.

## 4. A Qualitative Comparison between Model Predictions and Literature Experimental Results

The thermal stability of nanostructured solid solutions is typically investigated by annealing the as-produced material at different temperatures for different times. This way, conditions for which grain growth is suppressed and significant precipitation or local ordering do not take place can be identified. However, the interpretation of experimental results is not straightforward. In fact, depending on the adopted operating conditions, polycrystalline materials can be apparently stable, even though they do not represent the favorite state from the thermodynamic point of view. For instance, coarse microstructure at low temperatures may appear as the material stable state in place of the single crystal one because of the slow diffusional processes. Moreover, materials where grain boundary mobility is characterized by high activation energies may show coarsening resistance and be identified as thermally stable. It is then obvious that, from a thermodynamic perspective, the (apparent) kinetic stability just described is a transient state whose (apparent) stationarity is a consequence of the adopted observation time scale shorter than the system evolution characteristic time. Indeed, the latter one can be extremely long at low temperature due to the slow diffusion phenomena or in the case of drag forces exerted on grain boundaries, which can dramatically reduce GB mobility to virtually immobilize them.

According to the descriptions that are reported above, even though kinetic and thermodynamic stabilities are intrinsically different, their experimental assessment, identification, and results interpretation present several difficulties. Moreover, the interplay between thermodynamic and kinetic stabilization mechanisms has not been explored in detail so far, so that the conditions under which each mechanism dominates and whether the two act together or compete are yet to identify. Subsequently, a thorough experimental confirmation of the proposed model needs a specific and dedicated investigation, which will be addressed in a future paper. However, a preliminary qualitative comparison between model predictions and properly selected experimental findings that are taken from the literature for the case of W-Cr alloys are provided in what follows.

A supersaturated solid solution of W-15 at.% Cr was obtained through mechanical alloying [27]. As-milled powders had an average grain size of about 13 nm after 20 h of milling. Therein, it was shown that high-energy milling allows W and Cr atoms to be homogeneously distributed over the sample without any evident phase separation or chemical partitioning. However, it was found that this structure evolves upon heating with nanosized Cr precipitates starting to emerge near 950 °C. However, further annealing of the specimen up to 1400 °C resulted in the dispersion of Cr into the W-rich grains, thus moving the system toward a single nanostructured solid solution with a grain size of about 40–50 nm. Under these conditions, the existence of Cr segregation at grain boundaries was also verified. These results were interpreted by the authors as a clue of the possible thermodynamic stability of the nanocrystalline W-Cr alloy at 1400 °C.

It was shown in the previous section that a critical value of the interaction energy in the grain boundary, $\Omega^{*}$, exists, such that the condition $\Omega^{\left(gb\right)}<\Omega^{*}$ can be regarded as a criterion for thermodynamic stability assessment. The application of the proposed procedure to the W-Cr system at 1400 °C gives $\Omega^{*}=-1.01\;\mathrm{kJ}/\mathrm{mol}$ ($\beta^{*}=-0.031)$. In order to validate the model prediction, the value of $\Omega^{\left(gb\right)}$ needs to be necessarily estimated by other methods. Unfortunately, as already specified, a widely accepted and reliable methods to estimate $\Omega^{\left(gb\right)}$ are not yet available in the literature. However, a rough estimation can be obtained by taking advantage of the concept of enthalpy of segregation. The latter quantity can be related to the interaction energy in the grain boundary, as follows [6]:

(28) $${\Delta H}_{seg}=\left(\Omega^{\left(b\right)}-\Omega^{\left(gb\right)}\right)+{\hat{a}}_{A}^{0}\sigma_{A}^{\left(gb\right)}-{\hat{a}}_{B}^{0}\sigma_{B}^{\left(gb\right)}$$

(Note that Equation (28) has been obtained by setting the parameter υ appearing in Equation (25a) of the reference [6] equals to zero; this is for consistency of Equation (13), which does not consider the transitional zone originally proposed in the same paper). Using Equation (28) with ${\Delta H}_{seg}=52\;\mathrm{kJ}/\mathrm{mol}$ [15] along with other parameters value taken from Table 1, it can be easily estimated $\Omega^{\left(gb\right)}=-12.05\;\mathrm{kJ}/\mathrm{mol}$. This value satisfies the proposed criterion so that the thermodynamic stability of polycrystalline W-15 at.% Cr solid solution at 1400 °C is predicted.

**Figure 4:**
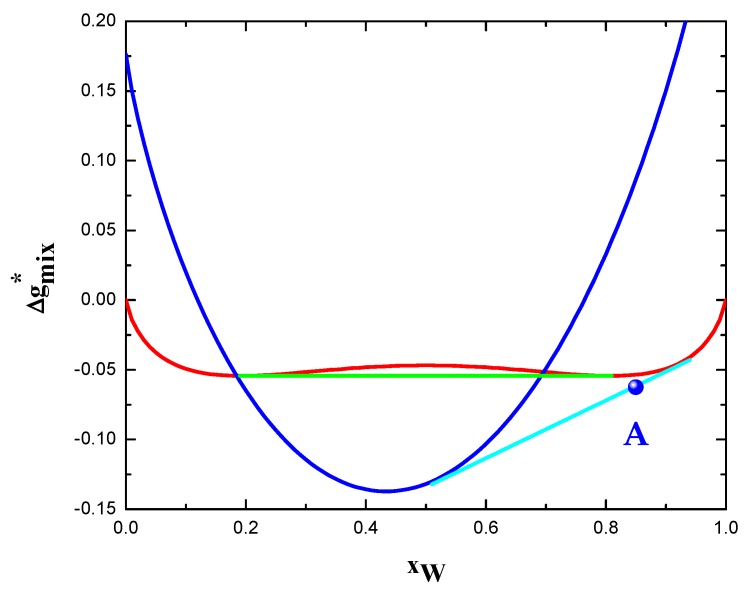
Phase equilibria of the system W-Cr at 1673.15 K. Dimensionless Gibbs free energy as function of composition is reported for bulk solid solution (red), phase separated state (green), as well as grain boundary phase (blue). Polycrystalline stable states are given by the common tangent (cyan) between grain boundary phase and bulk solid solution. Point A represents the most stable state of the experimentally investigated W-15 at.% alloy.

The qualitative agreement between model predictions and experimental findings can be also seen in Figure 4. The most stable state of W-15 at.% Cr at 1400 °C is represented by the polycrystalline solid solution (point A). The composition of grain interior and grain boundary are approximately $x_{Cr}^{\left(g\right)}=0.06$ and $x_{Cr}^{\left(gb\right)}=0.51$, respectively. These values are qualitatively in agreement with the experimentally observed grain boundary segregation of chromium [27]. Applying the level rule in Figure 4, the calculated GB atomic fraction results about 0.21. Grain size can be roughly estimated through Equation (18) by assuming that the partial molar volumes of alloy components are all equal in both phases (see Equation (17)). Afterwards, using $f^{\left(gb\right)}=0.21$ and t=5 nm [27], a grain size of 66 nm can be calculated. This value is not too distant from the one experimental measured, i.e., 40–50 nm [27].

As a concluding remark, it is worth pointing out that the proposed model should be considered as a first attempt to extend the theoretical investigation on thermodynamic stability to non-dilute polycrystalline alloys without any restriction concerning the materials grain size, the sign of the interaction energy (i.e., immiscible or miscible metals), and in the case of intermetallics-forming systems. The applicability of the model is strongly dependent on the availability of GB interaction energy values, as well as reliable experimental data shedding some light on the interplay between thermodynamic and kinetic stabilization mechanisms. In addition, a future work will address the effect of vacancies and dislocations to the GB structure.

## 5. Conclusions

A thermodynamic model of general application (e.g., without the assumption of dilute solutions or the limitation to immiscible systems) is formulated. The derivation is independent of structure geometry and it includes the interaction among grain boundary atoms as a peculiar feature. A methodology for the theoretical assessment of the thermodynamic stability of polycrystalline alloys according to the phase equilibria scheme is also proposed. This way, stability against grain growth, as well as against phase separation and intermetallic compound formation, can be predicted. The resulting theoretical tools is applied to W-based systems and a comparative analysis of various binary alloys is provided. It is shown that a critical value $\Omega^{*}$ of the interaction energy in the grain boundary $\Omega^{\left(gb\right)}$ exists, such that the condition $\Omega^{\left(gb\right)}<\Omega^{*}$ can be regarded as a criterion for thermodynamic stability assessment. The critical value of interaction energy should simultaneously satisfy the following conditions: $\Omega^{*}<\Omega^{\left(b\right)}$ and $\Omega^{*}<0$. The latter implies that a negative interaction energy in the grain boundary is a necessary condition for the thermodynamic stabilization of binary polycrystalline metallic alloys. Notably, the results that are presented in this work seem not to confirm the expectation often reported in the literature that thermodynamic stability of nanocrystalline metallic alloys is more plausible for immiscible systems. Model predictions are qualitatively validated through a preliminary comparison with experimental results being taken from the literature.

## Figures and Tables

**Table 1:** Parameters used in this work (All values are in kJ/mol).

Solute (B)	$\Delta {\hat{h}}_{B\to A}^{\left(chem\right)}$	$\Delta {\hat{h}}_{B\to A}^{\left(elast\right)}$	$\Delta E^{REF}$	${\hat{a}}_{i}\sigma_{i}^{\left(gb\right)}$	Solute (B)	$\Delta {\hat{h}}_{B\to A}^{\left(chem\right)}$	$\Delta {\hat{h}}_{B\to A}^{\left(elast\right)}$	$\Delta E^{REF}$	${\hat{a}}_{i}\sigma_{i}^{\left(gb\right)}$
**Li**	168.43	5.09	−1.04	2.72	**Tc**	−27.40	5.46	−0.15	12.44
**Be**	−17.63	113.07	−6.89	7.31	**Ru**	−38.06	12.59	−1.19	11.64
**Na**	390.12	28.64	5.57	2.02	**Rh**	−35.52	10.08	−0.32	10.39
**Mg**	143.39	22.41	0.02	4.14	**Pd**	−25.80	2.20	1.55	8.26
**Al**	−53.47	0.55	−1.29	5.05	**Ag**	169.62	1.65	2.96	5.53
**K**	599.01	45.18	9.56	1.56	**Cd**	100.39	18.32	5.72	3.84
**Ca**	262.10	78.34	2.67	4.06	**In**	117.85	44.69	7.46	3.98
**Sc**	38.34	48.57	0.60	7.29	**Sn**	61.99	117.55	5.53	4.07
**Ti**	−22.41	3.60	−0.57	9.50	**Sb**	45.63	55.94	3.85	3.31
**V**	−2.97	7.19	−0.52	9.86	**Cs**	709.21	52.15	15.69	1.50
**Cr**	3.46	31.73	−2.71	8.07	**Ba**	382.32	108.80	8.91	3.93
**Mn**	22.85	14.24	−0.12	5.68	**La**	154.18	86.68	7.24	7.64
**Fe**	−0.21	34.02	−7.88	8.57	**Hf**	−27.27	44.65	3.26	11.41
**Co**	−5.07	49.65	−7.19	8.50	**W**	-	-	-	15.52
**Ni**	−11.32	52.92	−1.72	8.09	**Ta**	−29.54	7.87	2.64	14.45
**Cu**	79.37	29.18	0.16	6.34	**Re**	−16.27	3.47	1.15	14.45
**Zn**	28.09	0.36	2.69	4.07	**Os**	−37.82	9.03	0.01	13.43
**Ga**	−3.99	10.12	2.42	5.36	**Ir**	−61.60	7.49	0.86	11.74
**Ge**	−29.75	0.29	−0.46	4.32	**Pt**	−81.44	1.27	2.69	10.12
**Rb**	648.37	60.53	13.17	1.51	**Au**	47.87	2.00	4.43	6.62
**Sr**	349.08	100.17	6.88	4.03	**Hg**	127.69	18.82	12.89	3.15
**Y**	110.41	90.61	3.63	7.75	**Tl**	189.52	56.03	9.45	3.60
**Zr**	−38.59	46.10	1.96	10.90	**Pb**	175.90	80.73	9.60	3.91
**Nb**	−33.39	7.09	1.09	12.38	**Bi**	152.68	72.25	7.19	3.31
**Mo**	−0.87	0.14	−1.21	12.54	**Po**	−12.72	35.45	0.00	3.02
